# P-106 - WASTEWATER BASED EPIDEMIOLOGICAL SURVEILLANCE FOR ANTIMICROBIAL RESISTANCE AND INFECTIOUS DISEASE: STRENGTHENING REAL-TIME POPULATION LEVEL PREPAREDNESS FOR THE ISLE OF MAN

**DOI:** 10.1093/pubmed/fdag046.097

**Published:** 2026-07-09

**Authors:** Ms Theresa Thornton, Dr Rebecca Rowley, Dr Matthew Tyrer

**Affiliations:** Public Health Isle of Man; Public Health Isle of Man; Public Health Isle of Man

## Abstract

**Background:**

The Isle of Man’s natural island border provides a unique setting to assess the local dynamics of infectious disease and antimicrobial resistance (AMR). A previous wastewater based epidemiological (WBE) pilot, conducted from March 2022 to July 2023, demonstrated the feasibility of population-level passive surveillance for five high-burden pathogens using bait-capture sequencing. Alongside this, the Island maintains established hospital-based AMR surveillance, accredited through the Global Antimicrobial Stewardship Accreditation Scheme (GAMSAS), and clinical infectious disease surveillance reporting across primary and secondary care. However, these clinical systems rely on healthcare-seeking behaviour and diagnostic pathways, limiting timely insights into community transmission. Developing an enhanced, integrated surveillance programme within this closed population is therefore novel and offers an opportunity to strengthen real-time population-level preparedness.

**Objectives:**

- Design a scalable WBE programme that complements existing infectious disease and AMR surveillance and embeds strategic risk assessment in interpretation and response.
- Improve situational awareness and support prioritised public health action.
- Enhance antimicrobial stewardship within a One Health framework.

**Methods:**

Building on the 2022 pilot, this project is evaluating analytical approaches for integrated AMR and infectious disease surveillance and defining pathways for alignment with current public health systems. Planned WBE targets include SARS-CoV-2, influenza A, norovirus, enterovirus and rotavirus, as well as priority AMR markers aligned with neighbouring jurisdictions. A single sampling site serving a large proportion of the population enables broad coverage while maintaining operational simplicity and cost-effectiveness.

**Results:**

The programme will deliver timely population-level pathogen and AMR insights, strengthening early detection, guiding rapid public health action, and enhancing long-term infectious disease preparedness and AMR stewardship.

**Conclusions:**

WWES provides a cost-effective, equitable and complementary approach to strengthening public health surveillance on the Isle of Man. Leveraging existing infrastructure, the programme will deliver actionable community-level data to support antimicrobial stewardship, enhance infectious disease preparedness and build long-term health protection resilience.

